# Attitudes of women towards screening, prevention and treatment of cervical cancer in Namibia

**DOI:** 10.4102/hsag.v29i0.2433

**Published:** 2024-02-01

**Authors:** Epafras Anyolo, Kristofina Amakali, Hans J. Amukugo

**Affiliations:** 1 Department of General Nursing Science, Faculty of Health Science and Veterinary Medicines, University of Namibia, Windhoek, Namibia

**Keywords:** knowledge, attitude, practices, screening, prevention, treatment, cervical cancer, reproductive women

## Abstract

**Background:**

Cervical cancer is among the top causes of cancer-related deaths among women globally.

**Aim:**

The aim of the study was to assess the attitudes of women of reproductive age towards cervical cancer services in the Otjozondjupa region, Namibia.

**Setting:**

The setting of this study was the Otjozondjupa region, one of the 14 regions of Namibia.

**Methods:**

A quantitative, cross-sectional, descriptive study was used. The data were collected with a questionnaire from a stratified sample of 381 out of the 37066 study population of women of reproductive age across four districts of the region. Descriptive data analysis was performed using Statistical Package for Social Science version 25.

**Results:**

The findings revealed that most respondents (83.1%) had an overall neutral attitude towards the screening, prevention, and treatment of cervical cancer; they were not concerned about the risk of cervical cancer and would not practice health-seeking behaviours regarding cervical cancer services.

**Conclusions:**

The respondents were indifferent with regard to prevention, screening, and management services for cervical cancer, implying that they would not use available cervical cancer services.

**Contributions:**

Attitudes of women of reproductive age towards cervical cancer services were highlighted. Subsequently, an educational programme for nurses to enhance the utilisation of cervical cancer services among women of reproductive age in the Otjozondjupa region, Namibia, was developed. Guidelines were developed to facilitate the implementation and evaluation of the educational programme activities to enhance the utilisation of cervical cancer screening services among women of reproductive age.

## Introduction

Cervical cancer is among the leading causes of gynaecologic cancer-related deaths in low- and middle-income countries (World Health Organization [WHO] 2020a). However, since the introduction of the Pap smear in 1941 and subsequent effective screening and treatment programmes, both the incidences and mortality of cervical cancer have markedly decreased.

Nevertheless, the uptake of screening for prevention and treatment services for cervical cancer in Namibia is reported to be low (WHO 2014). Furthermore, the annual cases of cervical cancer and deaths from cervical cancer in Namibia have been estimated at 2200 and 1238, respectively WHO 2020b. Cultural diversity and socio-economic aspects influence women’s knowledge, attitudes, and practice towards screening for the prevention and treatment of cervical cancer (Lim & Ojo 2017; Ndejjo et al. 2016; WHO 2019).

Attitudes play an important role in formulating health-seeking behaviour (Jassim, Obeid & Al Nasheet 2018; Kei et al. 2016). Concurrently, a negative attitude is associated with low uptake of screening services for the prevention and treatment of cervical cancer among women in sub-Saharan Africa (Jassim et al. 2018; Lim & Ojo 2017; Makurirofa et al. 2019), which may also apply to women in the Otjozondjupa region in Namibia, the setting of this study. Moreover, the data on screening for cervical cancer in the Otjozondjupa region indicate low uptake for screening, prevention, and treatment services for cervical cancer (Ministry of Health and Social Services) (MoHSS 2016).

The Health Belief Model postulates that one would engage in health-seeking behaviour if He, or she perceives the benefits that may be accrued from the recommended behaviour, such as attending cervical screening services (Glanz et al. 2015). Therefore, attitude is a determinant factor of women’s health-seeking behaviour towards cervical cancer.

Women from the Otjozondjupa region in Namibia are characterised by diverse socio-economic qualities based on their level of education and cultural practices, which may influence their attitude towards screening for cervical cancer (MoHSS 2016). This article presents the findings on the attitudes of women of reproductive age towards the screening, prevention, and treatment of cervical cancer in the Otjozondjupa region of Namibia.

## Aims and objectives

The literature indicates that cultural diversity and socio-economic factors such as fear of the screening procedure, fear of a positive result, reservation for potential exposure to a male healthcare provider, and financial insecurity are attitudinal barriers to women’s access to cervical screening services (Carvalho 2016; Msyamboza et al. 2016; Pittalis et al. 2020). Subsequently, the Namibia Demographic Health Survey (DHS) of 2013 reports that nationally, only 23.6% of women used screening services for cervical cancer. Specific to the Otjozondjupa region, only 0.29% of women of reproductive age had cervical cancer screening in 2014 (MoHSS 2014). Moreover, there was no record of cases using cervical cancer services in the Tsumkwe constituency of the Otjozondjupa region for the period 2015–2016 (MoHSS 2015/2016), implying that most of the women of reproductive age in Otjozondjupa region do not attend the services for cervical cancer.

Low national and local response to cervical cancer services is presumably because of inadequate knowledge and negative attitudes towards cervical cancer services among women of reproductive age in Namibia (CAN 2014; MoHSS 2014). Therefore, this study aimed to describe the attitudes of women of reproductive age in Otjozondjupa towards cervical cancer services.

### Aim of the study

The aim of the study was to determine the attitude of women of reproductive age regarding screening, prevention, and treatment of cervical cancer as the basis for the development of the education programme to enhance the utilisation of cervical cancer services among women of reproductive age in Otjozondjupa region, Namibia.

### Objectives of the study

The specific objectives of the study were to determine and describe the attitude of women of reproductive age in the Otjozondjupa region regarding screening, prevention, and treatment of cervical cancer.

## Research design and methods

### Research design

The study used a quantitative approach, a cross-sectional design and descriptive methods to quantify and describe the attitude of women of reproductive age regarding screening, prevention, and treatment for cervical cancer and to describe the association between variables from the data collected at one point in time.

### Study setting

The setting of this study was the four health districts of the Otjozondjupa region in Namibia. Screening for cervical cancer takes place at clinics and health centres.

### Study population and sampling strategy

The study population comprised 37 066 women of reproductive age in all four districts of the Otjozondjupa region. Firstly, each district was designated as a stratum for each population element to belong to a stratum. All four districts were included in the study as strata. Secondly, 3 health centres and 15 clinics were identified as strata within the four districts. Thirdly, the simple random sampling technique was used to select the health centres and clinics in each stratum. Three health centres and 9 clinics were randomly selected and participated in the 12 studies as strata. A sample size was proportionally calculated from each stratum at a confidence interval of 95% and a margin error of 5% (size [n] = N/1+Ne^2)^ for each of the 12 strata (Brink et al. 2012). A proportionate multistage stratified random sampling was used to ensure all the segments were proportionately represented. A proportional, random selection of participants was performed at each health facility. A total of 90 participants were randomly selected from the health centres and a total of 291 participants were randomly selected from the clinics, with a final sample size of 381 participants (Table 1); however, the study sample size was purposefully increased to 400 by adding 9 more respondents to the calculated sample size in order to enrich the representativeness of the findings.

**TABLE 1:** Study population and sample size.

District	Population of women	Proportional sample size
Grootfontein	10 506	108
Okahandja	7230	74
Okakarara	6117	63
Otjiwarongo	13 213	136
Region	37 066	381

*Source:* Ministry of Health and Social Service (MoHSS), 2016, Annual report (2015-2016), Republic of Namibia, Windhoek, Namibia

### Data collection

#### Research instrument

The data were collected with a questionnaire, either self-administered or researcher-administered adopted from the literature regarding women’s attitudes towards cervical cancer services, to ensure construct validity (Meyer & Van Niekerk 2008). The questionnaire was prepared in English, and the researcher could interpret the questionnaire to the respondents as he is conversant with most local languages spoken at the study sites. The questionnaire consisted of two sections. Section A focused on the respondents’ demographic data. Section B obtained data concerning the respondents’ attitudes towards cervical cancer services regarding the screening, prevention, and treatment.

The questionnaire contained close-ended questions of dichotomous ‘Yes’ and ‘No’ responses and Likert scale with five options of responses: strongly agree, agree, neutral, disagree, strongly disagree, and ‘do not know’ about respondents’ attitudes towards prevention, screening, and the treatment for cervical cancer. All statements were scored, summed up, and categorised as negative, neural, and positive. A Cronbach’s alpha test was calculated to evaluate the internal consistency and reliability of the findings and yielded alpha values between 0.828 and 0.902 for knowledge variables, while variables on cultural beliefs yielded a value of 0.573 and these values indicated that the data-collection instrument was reliable and consistent (Cortina 1993).

#### Data collection procedure

The researcher obtained access to the research sites from the site managers. The respondents were accessed in the waiting area as they came for various services. The purpose and objectives of the study were explained, and informed consent was obtained from the respondents. Data were collected from the respondents in the room arranged at each health facility. Respondents who could read and write completed the self-administered questionnaire, while data from the respondents who could not read and write were collected through the researcher-administered interviews.

To ensure anonymity in the study, the respondents were identified with codes. Completed questionnaires were placed in the box at the study site. Completion of a questionnaire took 30–35 min. The data-collection process started on 03 July 2019 and ended on 15 September 2019.

#### Data analysis

A total of 400 questionnaires were completed; however, 6 (1.5%) of the 400 questionnaires were incomplete, thus yielding a study’s response rate of 98.5%. The 394 questionnaires were cleaned and processed for analysis.

A data entry sheet in Microsoft Excel was created to capture the data and then imported into the statistical package for Social Science (SPSS) version 25 for analyses. Descriptive statistics were performed to measure the means, median, and standard deviation. Chi-squared tests and Pearson correlations (r) were used to determine the associations between dependent and independent variables. A *p*-value less than 0.05% was considered statistically significant. Frequency distribution, and proportions, were used to present the findings about respondents’ demographic data and attitudes towards cervical cancer services in tables, figures, and graphs.

### Ethical considerations

This research was conducted to fulfil the requirement for the completion of the Doctoral Degree in Nursing Science. The study was approved by the Human Research and Ethical Committee (HREC) of the University of Namibia and an Ethical Clearance Certificate was granted. Ethical approval and permission to conduct the study at health facilities were granted by the MoHSS. The aim and objectives of the study were explained to the respondents, participation was voluntary, and the respondents had the right to withdraw at any time without repercussions. Anonymity was ensured and confidentiality of the information was maintained.

## Results

Out of a total 400 questionnaires, 394 questionnaires were completed, and yielded (98.5%) study response.

### Demographic data of respondents

The demographic data on variables of age, marital status, educational level, employment status, home language, duration of stay in the area, and parity of the respondents were obtained.

### Attitude regarding prevention, screening, and treatment of cervical cancer

The study determined the attitude of women of reproductive age with regard to the prevention, screening, and treatment of cervical cancer findings of which are discussed further in the text.

### Attitude regarding the prevention of cervical cancer

Three items determine respondents’ attitudes on psychosocial influences on preventing cervical cancer.

#### Views whether cervical cancer is real and dangerous

Sixty-nine (17.5%) strongly agree, 133 (33.8%) agreed that cervical cancer is real and dangerous. Furthermore, 159 (40.4%) had a neutral attitude concerning the reality and danger of cervical cancer, while 23 (5.8%) of the respondents did not know that cervical cancer is real.

#### Perception of being at risk for cervical cancer

A total of 40.6 % of the respondents perceived themselves as not at risk for cervical cancer. Slightly less than 30% of the respondents, 116 (29.5%), did not know whether they were at risk of cervical cancer, while 45 (11.5%) were neutral.

#### Consultation of the doctor or a nurse in case of abnormal vaginal bleeding between the periods

About 43.7% of the respondents were neutral about whether to consult the doctor or a nurse in case of abnormal vaginal bleeding between their periods, 123 (31.5%) did not know, and only 16.9% affirmed that they would go to the health facility to consult the doctor or nurse.

### Attitude regarding screening for cervical cancer

Five items were assessed to determine respondents’ attitudes regarding psychosocial influences on the screening for cervical cancer.

#### Consultation with a family member about cervical cancer

The majority of the respondents, 210 (54.7%), did not know, about 129 (33.6%) 49 disagreed, only 13 (3.4%) agreed, and the rest 32 (8.3%) of the respondents were neutral about consultation of a family member before deciding to screen for cervical cancer.

#### Discussion with the spouse before deciding to screen for cervical cancer

Only 18 (4.6%) of the respondents affirmed that they will discuss their decision to go for cervical cancer screening with their spouse. The majority of the respondents, 203 (51.8%), did not know. In comparison, a total of 119 (30.4%) disagreed and strongly disagreed, and 52 (13.3%) were neutral whether to discuss with their spouses before deciding to screen for cervical cancer.

#### Fear of a positive cervical cancer result

Most of the respondents, 153 (39.5%), indicated fear of a positive cervical cancer result; of the respondents, significant 133 (34.4%) were neutral, while 69 (17.8%) did not know.

#### Fear and shame for the examination procedure

Most of the respondents, 173 (44%), indicated that they feared and would be ashamed to undergo a cervical cancer screening procedure. A further 125 (31.7%) were neutral, 47 (12%) did not fear and would not be ashamed, and 49 (12.4%) did not know about their feelings concerning cervical cancer screening.

#### A lack of time to go for cervical cancer screening because of other responsibilities

Of the respondents, 63 (16.0%) indicated that lack of time prevents them from seeking cervical cancer screening services, while 147 (37.3%) were neutral.

Table 2 displays the findings about the overall level of attitudes towards cervical cancer owing to psychosocial influences on screening for cervical cancer.

**TABLE 2:** The overall level of attitude because of psychosocial influences on cervical cancer.

Scores on the level of attitude	Frequency	Percent	Valid percent	Cumulative percent
**Valid**				
Negative attitude	126	32.0	34.1	34.1
Neutral attitude	223	56.6	60.3	94.3
Positive attitude	21	5.3	5.7	100.0
Total	370	93.9	100.0	-
**Missing**				
System	24	6.1	-	-
Total	394	100.0	-	-

*Source:* Anyolo, E., 2023, *Development of an educational programme for enhance the utilization of cervical cancer services among women of reproductive age in Otjozondjupa region*, Dissertation, University of Namibia, Windhoek, Namibia

### Cultural beliefs on the screening for cervical cancer

Five items related to cultural beliefs that could influence women’s attitudes towards screening for cervical cancer were assessed.

#### Afraid of being screened by a known healthcare provider

Close to half of the respondents, 187 (47.6%), were neutral on whether they are afraid of being screened by known healthcare providers, while a total of 145 (36.9%) were not afraid. However, 45 (13.7%) were afraid to be screened by a known healthcare provider.

#### Shame to be associated with cervical cancer disease

The majority of the respondents, 219 (55.6%), were neutral, a few 71 (18%) were ashamed, and only 95 (14.1%) would not be ashamed to be associated with cervical cancer.

#### Shame to be screened by a male health service provider

The majority, 283 (71.8%%) of respondents, indicated that they felt ashamed to be screened by male health service providers, while 56 (14.2%) were neutral and only 24 (6.1%) stated that they felt no shame to be screened by male health service providers.

#### Shame to be screened by a young health service provider

The majority of the respondents, 164 (41.8%), positively affirmed that they would not be ashamed to be screened by a youthful healthcare provider. Of the respondents, 103 (26.3%) agreed that they do not want to be screened by a youthful healthcare provider, while 122 (31.1%) were neutral about being screened by a youthful health service provider.

#### Intrusiveness of the procedure

A total of 141 (36%) of the respondents indicated that the procedure is too intrusive. However, 126 (32.2%) of respondents perceived the procedure not to be intrusive while 109 (27.8%) were neutral, saying that the procedure was too intrusive.

Table 3 displays the findings about the influences of culture towards screening for cervical cancer.

**TABLE 3:** The overall level of attitude because of cultural beliefs on the screening of cervical cancer.

Scores on the level of attitude	Frequency	Percent	Valid percent	Cumulative percent
**Valid**				
Positive Attitude	14	3.6	3.6	3.6
Neutral Attitude	225	57.1	57.8	61.4
Negative Attitude	150	38.1	38.6	100.0
Total	389	98.7	100.0	-
**Missing**				
System	5	1.3	-	-
Total	394	100.0	-	-

*Source:* Anyolo, E., 2023, *Development of an educational programme for enhance the utilization of cervical cancer services among women of reproductive age in Otjozondjupa region*, Dissertation, University of Namibia, Windhoek, Namibia

### Personal factors that influence screening for cervical cancer

Six items about personal factors influencing cervical cancer screening among women were assessed.

#### Shyness to go for screening

Many of the respondents, 199 (51.0%), were neutral if they would be shy to go for screening; a significant 111 (28.4%) felt shy to go for screening. Only 61 (15.7%) would not be shy, while 19 (4.9%) did not know.

#### Fear of embarrassment from screening

Most of the respondents, 234 (76.7%), were neutral, 93 (23.2%) feared embarrassment, 45 (23.5%) did not fear embarrassment, and 21 (5.4%) were silent about embarrassment as a result of undergoing the Pap smear procedure.

#### Fear of the test results

Most of the respondents, 218 (55.7%), were neutral about the fear of getting the test results, and 121 (30.8%) feared to hear the results. Only a few, 31 (13.2%), would not fear, while 19 (4.8%) of the respondents did not know.

#### Feeling not at risk because of not being sexually active

Close to half, 191 (48.8%), of the respondents submitted that they are not at risk for cervical cancer because they were not sexually active. Only 43 (11%) felt being at risk. However, 128 (32.7%) were neutral, and 29 (7.4%) did not know if they were at risk of cervical cancer.

#### Do not suffer of any symptoms

The majority, 178 (45.4%), of the respondents indicated that they would not seek screening if they do not suffer from symptoms suggestive of cervical cancer, 126 (32%) were neutral. Only a few, 35 (8.9%), would seek screening even if they do not suffer from symptoms suggestive of cervical cancer, while 53 (1.5%) did not indicate their opinion.

#### Awareness of availability of screening services

Of the respondents, 159 (40.6%) were aware of the availability of the screening services, 160 (40.8%) were neutral, 49 (12.5%) were not aware, while 24 (6.1%) could not indicate whether they were aware or not aware of the availability of the cancer-screening services.

### Economic factors that influence the uptake of cervical cancer screening

Five items related to the economic factors likely to hinder women from utilising cervical cancer screening services were assessed.

#### Distance from home to the health facility

All respondents knew the distance between their residence and health facilities for cervical cancer screening services. A significant 116 (29%) of the respondents lived less than 1 km from a health facility. Of those who lived within 5 km – 10 km, 76% indicated the distance to healthcare facilities hinders them from seeking cervical cancer screening services.

#### A lack of transport from home to the facility and the cost involved

Most respondents (90.4%) indicated a lack of transportation from their homes to the facility, while 90.8% indicated that the transport cost was not affordable.

#### Attitude regarding treatment for cervical cancer and chances of recovery

Most of the respondents, 131 (33.3%), reported that they did not know that cervical cancer can be treated if detected early, 94 (23.9%) disagreed, only 69 (17.6%) knew that cervical cancer can be treated if detected early, while 56 (14.2%) were neutral.

#### Overall attitudes regarding cervical cancer

With regard to attitudes towards cervical cancer screening, the majority of the respondents, 304 (83.1%), had a neutral attitude, 47 (12.8%) had a negative attitude, and only 15 (4.1%) of the respondents had a positive attitude towards cervical cancer services. Table 4 displays the overall score for the respondents’ attitudes regarding cervical cancer screening services.

**TABLE 4:** The overall attitude regarding cervical cancer.

Scores on the level of attitude	Frequency	Percent	Valid percent	Cumulative percent
**Valid**				
Negative attitude	47	11.9	12.8	12.8
Neutral attitude	304	77.2	83.1	95.9
Positive attitude	15	3.8	4.1	100.0
Total	366	92.9	100.0	-
**Missing**				
System	28	7.1	-	-
Total	394	100.0	-	-

*Source:* Anyolo, E., 2023, *Development of an educational programme for enhance the utilization of cervical cancer services among women of reproductive age in Otjozondjupa region*, Dissertation, University of Namibia, Windhoek, Namibia

## Discussion

Age is a factor for accessing cervical cancer screening services. The findings of this study indicated that 22.1% of the respondents were within the 30 years – 39 years age bracket, where cervical cancer is the most common cause of morbidity and mortality, and accounts for 16% of all cancers diagnosed in this age group (WHO 2017). In congruence with these results, other studies have revealed that women who often uptake screening services for cervical cancer tend to be younger (aged 30–39), which is similar to the 22.1% respondents of this study (Mabelele et al. 2018; Makurirofa et al. 2019). Thus, based on the findings of this study, health information about screening, prevention and treatment for cervical cancer in Namibia should target the age group from 20 years and above to empower them with the knowledge and a positive attitude to take up cervical screening services.

Over half (58.1%) of the respondents were single, followed by married respondents (28.1%). Co-habiting women amounted to 9.8% of the respondents. Similar trends were observed in Kenya, Zambia, and Zimbabwe (Lim & Ojo 2017; Makurirofa et al. 2019).

Level of education also plays a role regarding access and utilisation of cancer screening services. The findings indicated a cumulative 79% of the respondents have obtained secondary and tertiary education, indicating they are literate and compatible with health information regarding preventative health services for cervical cancer. Various studies also observed that females who have formal education, have a higher likelihood of accessing screening services. In return, the level of knowledge would promote positive attitudes towards the uptake of cervical cancer screening services among women of reproductive age (Gatumo et al. 2018; Jassim et al. 2018).

Most respondents (52.8%) had one to –two births, while respondents with no births or three or more births were few. A previous study by Sankaranarayanan (2014) revealed that the number of childbirths directly correlates with the uptake of screening services, which implies that the majority of the respondents apply family planning information to control deliveries and if given health information about cervical cancer preventative health services, may use it too.

Most respondents (59.8%) resided in the study area for 6 years or more, while 28.0% had lived there for between 3 years and 5 years. These groups could know the availability of cervical cancer screening services at health facilities if healthcare providers disseminated cancer-preventative information at the point of care (Sudenga et al. 2013). Knowledge of health information would enable the development of positive attitudes towards cervical cancer health services.

Furthermore, the findings indicated that the home languages of all the respondents were vernaculars other than English, the official language. Health information about cervical cancer in Namibia is only available in English. Therefore, it was reported in this study that only 2.3% of the respondents could access Leaflets or brochures with health information on cervical cancer in local languages. In concurrence with the suggestion by Ndejjo et al. (2016), the findings call for the availability of brochures of health information on cervical cancer written in the vernaculars of the respondents of this study. This would make health information accessible to eligible clients. The demographic data of the respondents can be seen in Table 5.

**TABLE 5:** Demographic data of the respondents.

Variable	Frequencies and porportions	
	*n*	%
**Age in years**		
18–19	-	4.60
20–24	-	10.40
25–29	-	14.20
30–34	-	20.00
35–39	-	22.10
40–44	-	17.00
45–49	-	12.20
**Marital status**		
Single	225	58.14
Married	109	28.17
Divorced	3	0.78
Window	3	0.78
Separated	9	2.33
Cohabiting	38	9.82
**Educational level**		
Not attended school	43	10.94
Primary schooling	121	30.80
Secondary schooling	192	48.90
Tertiary	37	9.40
**Employment status**		
Students	18	4.50
Housewives	13	3.31
Employed	134	34.10
Self-employed	26	6.62
Unemployed	202	51.40
**Home languages**		
Herero	90	22.80
Damara-Nama	75	19.00
Oshiwambo	65	16.50
San	56	14.20
Kavango	39	9.50
Silozi	13	3.30
Others	25	6.60
**Length of stay in the study area in years**		
Less than 1 year	21	5.30
1–2 years	26	6.60
3–5 years	110	28.00
6 years and above	235	59.80
**Number of pregnancies**		
0 Birth	11	11.90
1–2	208	52.80
3 or more	139	35.30

*Source:* Anyolo, E., 2023, *Development of an educational programme for enhance the utilization of cervical cancer services among women of reproductive age in Otjozondjupa region*, Dissertation, University of Namibia, Windhoek, Namibia

The following sections present the discussions on attitude of women of reproductive age regarding the prevention, screening, and treatment of cervical cancer.

### Attitudes of women of reproductive age regarding the prevention and screening for cervical cancer

#### Prevention

Psychosocial factors determine whether women will seek cervical cancer prevention services. In accordance with the report by Mukama & Ndejjo (2017) that women often think that they are not at risk of cervical cancer, this study’s findings have proven likewise. In this regard, 43.2% of the respondents perceived themselves as not being at risk, and only 5.5% perceived as being at risk for cervical cancer. Furthermore, 40.4% of the respondents were neutral that cervical cancer is real. It is regrettable to observe that only a notable number of respondents perceived themselves as at risk of cervical cancer. Moreover, most of the respondents who perceived themselves as not at risk of cervical cancer were in the sexually active age brackets of 30 years – 34 years (18.2%) and 35 years – 39 years (23.3%) and were predisposed to the risks for cervical cancer. However, the Health Believe Model *postulates* that perceived severity and susceptibility to the disease guides the decision to seek a services such as cervical screening (Morema et al. 2014). Thus, dismissing the risk of cervical cancer among the respondents of this study hinders their health-seeking behaviours for cervical screening services. The WHO (2014, 2018) recommends that women of reproductive age between 30 years and 49 years and HIV negative should be screened for cervical cancer every third year, while those who are HIV positive should be screened every year to increase the chances of early detection of cervical cancer and ultimately recovery.

Thus, there is a need for health education programmes to sensitise women that all women of reproductive age are at risk of cervical cancer. Sensitisation would result in changes of the dismissal of vulnerability to cervical cancer. Individual perceptions of the problem, susceptibility, severity, and benefits would result in action to prevent cervical cancer.

Furthermore, among the majority (56.3%) who reported feeling at risk of cervical cancer, 57.7% had attained a secondary level of education, while 50.5% were employed women. The Pearson Chi-Square test revealed a positive relationship between attitude towards cervical cancer screening services and attainment of education and employment status (*p = <*0.01), respectively. Educated and employed respondents felt threatened by cervical cancer as they were likely to access and understand health information. Equally, they afford expenses to travel to and from health facilities for cervical cancer services. Concurrently, the literature indicates that being unemployed is one of the reasons most women do not access cervical cancer screening services and that they present late at healthcare facilities with invasive cervical cancer that could have been detected and treated early (Makurirofa et al. 2019).

The findings indicated that 43.7% of the respondents were neutral about whether to consult the doctor or a nurse in case of abnormal vaginal bleeding between their menstrual periods. In contrast, a similar study conducted in the Democratic Republic of Congo revealed different sentiments, such as that most respondents were willing to consult medical personnel in the case of abnormal bleeding between menstruations (Ali- Risasi et al. 2014).

#### Screening for cervical cancer

Partners play a major role in women taking up cervical screening. The study revealed that over 50% of the respondents do not know whether it is necessary to discuss the need to attend cervical screening with their spouses. These results are in accordance with the results from other studies, which revealed that the majority of male partners in sub-Saharan Africa do not permit their female partners to go for cervical screening, presumably because of fear of the violation of privacy through body exposure or because of suspicion about women being unfaithful to their male partners (Finocchaario-Kessler et al. 2016; Williams 2014). This implies that women may be afraid to discuss cervical screening with their spouses. Hence, there is a need for men also to be exposed to educational programmes about cervical cancer to become informed about the dangers of cervical cancer, the importance of preventive measures and curability if detected early, with their support being crucial in ensuring the health of their partners.

Receiving cervical screening results is a challenging moment for many women. The study revealed that most respondents (55.7%) were neutral about the fear of being told about a positive result of cervical cancer, while 34.4% feared positive results. Subsequently, over half of the respondents (55.6%) were neutral regarding feeling shame for being affected with cervical cancer. Similarly, different studies assert that individuals view of a positive result as a death sentence and thus see no point in going for screening (Gatumo et al. 2018; Jassim et al. 2018; Makurirofa et al. 2019). Solace in ignorance that ‘what you do not know cannot kill’ also causes a negative attitude towards cancer screening services. Therefore, an educational programme intervention should teach women that receiving a positive cervical screening result is a golden chance to access treatment and potential cure because cervical cancer detected at an early stage, such as the presence of pre-cancerous lesions, can be treated and cured. However, cervical screening is the only means to determine the onset of cervical cancer early.

The presence of male healthcare providers for the provision of reproductive health services is a significant barrier towards the uptake of cervical cancer screening. Similar to the findings by Gatumo et al. (2018) that women are often embarrassed to be attended by a male healthcare provider, this study revealed that the majority (72%) of the respondents indicated that the procedure is intrusive, embarrassing, and that they were ashamed of being screened by male health service providers. The provision of cervical cancer screening services by male healthcare providers is a major hindrance for most women to seek cervical cancer screening. The results call for the service providers to recruit female healthcare providers for cervical cancer screening services so that women can access screening services without reservation. Contrarily, this study indicated that a summative 34% of the respondents indicated that they are not ashamed to be provided cervical screening services by a male healthcare provider. These assertions imply that there is hope that women would attend the services, irrespective of the gender of the provider, if they are well-informed about the benefits of cervical screening.

Equally, and in support of the claim by Lim and Ojo (2017) that women in sub-Saharan Africa face stigmatisation and embarrassment when they receive health services from youthful healthcare providers, the current studies reported that 26.3% of respondents, irrespective of their age and educational level confirmed that they were ashamed of being screened by a young health service provider, while a notable 31.1% were neutral in that regard. Thus, the respondents considered youthful healthcare service providers to be barriers to the uptake of cervical screening. However, 41.8% of the respondents revealed they are not ashamed to be screened by young healthcare providers. Nevertheless, a report of respondents’ positive attitudes towards a young healthcare provider is encouraging. Therefore, educational programme interventions should educate women about adherence to ethical issues and assurance of confidentiality and privacy by healthcare providers, irrespective of their age.

Furthermore, the findings indicated that only a few (8.9%) of the respondents would seek cervical cancer screening services even if they do not experience symptoms. The majority were either neutral or opposed to seeking cervical cancer screening in the absence of the symptoms. In accordance with these results, similar studies report that women perceive no need for cervical screening until they present with symptoms (Kei et al. 2016; Mabelele et al. 2018 & Makurirofa et al. 2019). This study’s results clearly suggest an urgent need to educate women about the importance of routine and regular cervical screening because cervical cancer does not present symptoms in the early stage. Therefore, it is important to undergo screening for early detection to increase the probability of better treatment outcomes.

Economic factors are the main determinants for the uptake of cervical cancer screening services in many communities. Subsequently, 76% of the respondents who lived 5 km – 10 km away from the health facilities reported that long distances prevent them from accessing cervical cancer screening services. Consequently, 90.8% of the respondents indicated they could not afford the transport costs to travel to the health facilities. In congruence with this study’s findings, transportation costs to access services were also reported as a factor contributing to the low uptake of cervical cancer screening (Alemayehu & Mariam 2013; Gatumo et al. 2018). Unaffordable transport costs and long distances to the healthcare facilities might be the reason why 263 (68.0%) of the respondents in this study had never undergone cervical screening in their lifetime (Anyolo 2023). Therefore, screening services should be accessible to all eligible clients via mobile services irrespective of their economic status.

Regarding the respondents’ willingness to undergo screening for cervical cancer prevention, the findings indicated that the majority (89.8%) were willing to undergo cervical screening; of these, 81.9% preferred to have Pap smear tests at the local Primary Healthcare Clinic. Only 10% of the respondents were dismissive about taking cervical screening. Therefore, the primary healthcare facilities should be fully equipped with screening consumables and instruments and skilful healthcare workers to carry out the procedure.

#### Attitude regarding treatment for cervical cancer and chances of recovery

The findings revealed that 131 (33.3%) of the respondents did not know that cervical cancer can be treated if detected early. This is in concurrence with the respondents’ views of a positive result of cervical cancer screening as a death sentence, as they asserted that cervical cancer cannot be cured even if detected early. Similarly, various studies found that individuals view a positive result as a death sentence, and thus, they saw no point in going for screening (Gatumo et al. 2018; Jassim et al. 2018 & Makurirofa et al. 2019). Solace in ignorance that ‘what you do not know cannot kill’ also causes a negative attitude towards cancer screening services. In contrast, some studies revealed that most women believe cervical cancer can be treated and cured if detected earlier (Jassim et al. 2018; Touch & Oh 2018).

## Conclusions

The findings of this study concluded that the women of reproductive age in the Otjozondjupa region had attitudes ranging from neutral (83,1%) to negative (12.8%) and only 4.1% had positive attitudes towards the prevention, screening, and treatment services for cervical cancer. Therefore, appropriate and effective health educational programmes should address the psychosocial issues and cultural beliefs that act as barriers to cervical cancer (Anyolo 2023) screening services.

### Study limitations

The study was conducted in the Otjozondjupa region, one of the 14 regions in the country. Therefore, the findings may not be generalised to women of reproductive age in the other regions of the country. The instruments for data collection were in English and not translated into the vernacular of the respondents, and this might have an influence on the study results. However, the researcher conducted face-to-face interviews with the respondents through translations to overcome language barriers.
